# Electrically Evoked Itch in Human Subjects

**DOI:** 10.3389/fmed.2020.627617

**Published:** 2021-01-20

**Authors:** Hans Jürgen Solinski, Roman Rukwied

**Affiliations:** Department of Experimental Pain Research, Medical Faculty Mannheim, University of Heidelberg, Mannheim, Germany

**Keywords:** atopic dermatitis, rectangular pulses, sinusoidal stimulation, polymodal nociceptors, silent C-fibers, voltage-gated sodium channels, slow depolarization, peripheral (axonal) and central sensitization

## Abstract

Administration of chemicals (pruritogens) into the skin evokes itch based on signal transduction mechanisms that generate action potentials mainly in mechanically sensitive and *in*sensitive primary afferent C-fibers (pruriceptors). These signals from peripheral neurons are processed in spinal and supra-spinal centers of the central nervous system and finally generate the sensation of itch. Compared to chemical stimulation, electrical activation of pruriceptors would allow for better temporal control and thereby a more direct functional assessment of their activation. Here, we review the electrical stimulation paradigms which were used to evoke itch in humans in the past. We further evaluate recent attempts to explore electrically induced itch in atopic dermatitis patients. Possible mechanisms underlying successful pruritus generation in chronic itch patients by transdermal slowly depolarizing electrical stimulation are discussed.

## Text

Traditionally, basic researchers administered histamine into the skin of human subjects to experimentally evoke an itch response (pruritus). An alternative approach to induce itch is a depletion of histamine from skin mast cells by e.g., administration of codeine (1) or compound 48/80 (2). In chronic itch conditions, however, histamine is apparently not the major contributor driving chronic itch (3) and over the past decades a plethora of compounds (“pruritogens”) have been identified to cause histamine-independent itch. For example, the bovine adrenal medulla peptide 8–22 (BAM8-22), the anti-malarial drug chloroquine (CQ), the hexapeptide SLIGRL, but also endogenous substances such as cytokines (e.g., interleukine-31), proteases (e.g., trypsin), amino-acids (e.g., beta-alanine), peptide hormones (e.g., endothelin-1), and many others [for review see (4)]. These compounds act on their cognate receptors expressed on primary afferent neurons, for example Mas-related G protein-coupled receptors (Mrgprs) or protease-activated receptors (PARs) [e.g., see (5–7)]. Itch induced by these mediators involves signal transduction mechanisms that, in turn, induce the generation of action potentials (APs) in primary afferent neurons. Of these, unmyelinated nerve fibers—mechanically sensitive as well as mechanically *in*sensitive C-fibers—are instrumental for chemically evoked APs and their transmission to the central nervous system (CNS). This peripheral pruriceptive signal is processed in spinal and supra-spinal centers in the CNS to finally generate the sensation of pruritus (8, 9).

### Electrically Evoked Itch in Healthy Human Subjects

In order to improve temporal control and assess the function of peripheral itch-inducing nerve fibers more directly and thereby circumvent above mentioned chemical signal transduction mechanisms, their electrical activation was pursued for decades. In 1943, Bishop developed a constant voltage stimulator for repetitive electrical skin stimulation through a fine wire being in skin contact, and when combining weak electrical shocks with high stimulation frequency of about 10 Hz a pure sensation of itch could be evoked (10). He noted that the higher the frequency the lower the strength must be for the purest sensation of itch. Instead of a transcutaneous electrical stimulation, Shelley and Arthur inserted an electrode just beneath the epidermis and delivered biphasic square-waves of variable frequency, duration and intensity (11). The most robust pruritogenic effects were observed at a stimulation frequency of 20 Hz, a 5 ms pulse duration and a voltage ranging from 0.5 to 1.5 V. However, the current level could not be controlled sufficiently to avoid that cells and nerve fibers surrounding the electrode were immediately cauterized. Therefore, Edwards and co-workers used a solid state constant current generator placed between the square-wave generator and the subject, thus enabling the control of the current amplitude, and attached a transcutaneous electrode of 5 cm^2^ to the wrist of the subjects (12). The volar aspect of the wrist was chosen because at this site the greatest concentration of “itch points” was identified, i.e., areas at which an itch developed when stimulated electrically (11). Delivering mono-phasic repetitive current pulses of 50 Hz and 10 ms pulse duration induced reliable itch at threshold current levels of 15 μA and up to a maximum of 150 μA (12). On some occasions, subjects reported in addition to an itch a sensation of warmth or prickling, and the authors suggested a variation of the experimental procedure, for instance using bi-phasic rather than mono-phasic currents or other pulse forms than the delivered square-waves. Tuckett followed this suggestion, exploring the responses of feline “polymodal” nociceptors (mechanically- and heat-sensitive C-fibers) to different frequencies of electrical square-wave stimulation and correlating the results to the psychophysical assessment of electrical stimulation and application of cowhage spicules (*Mucuna pruriens*) in human subjects (13). One of his findings was that 10 Hz square-wave pulses of 7 ms duration and gradually increasing currents, delivered through 6 cm^2^ gauze pads placed to the wrist, induced pure itch in 50% of the subjects (in addition, about 40% of the subjects reported a mixture of itch and pain), which was very similar to the sensation from cowhage stimulation. While polymodal nociceptors tended to fatigue and their average response rate remained constant between 10 and 40 Hz, human subjects reported increased sensations with increasing frequency of stimulation, and the author suggested that increased pruritus at higher stimulation frequencies is signaled by few neurons that can follow higher frequencies of stimulation (13). About 15 years ago, Ikoma and colleagues modified aforementioned electrical stimulation protocols by delivering trains of 50 rectangular pulses (2 ms pulse duration) every 3 s for a period of 90 s across a 0.1 × 7 mm stainless steel wire attached to the wrist skin. Varying pulse frequency and current intensity revealed a maximum itch response at frequencies exceeding 50 Hz and current intensities lower than 0.12 mA (14). Higher current intensities frequently induced a tapping and pain sensation accompanied by a reduction of itch. The phenomenon of itch reduction by painful stimuli is most likely attributed to central inhibitory mechanisms (15, 16). A comparable method of itch induction comprised the use of a pair of disk electrodes with a diameter of 1 cm attached to the wrist (17) or the volar forearm skin (18) of human subjects. At both skin sites, itch was induced by delivering rectangular electrical pulses of 0.1 ms duration with 50 Hz and a current intensity that continuously increased over 2 min at 0.05 mA/s up to a maximum of 5 mA (wrist) or 6.4 mA (forearm).

All aforementioned studies suggest that itch can be induced by electrical stimulation in human skin. Of note, the parameters for electrical stimulation (current intensity, pulse frequency, stimulus duration and electrode configuration) need to be considered carefully as these determine which primary sensory afferent nerve fibers will be preferentially activated. As already mentioned, primarily unmyelinated C-nociceptors (named “pruriceptors”) are involved in itch signaling from the periphery to the CNS. Early recordings from the saphenous nerve of the cat provided evidence that the itching after-sensation to light touch results most probably from C-fiber activation (19). Isolation of small nerve fascicles and their separation into fine strands allowing for single-unit recordings of slowly conducting myelinated (20) and unmyelinated (21) neurons revealed that polymodal C-fibers most likely contribute to cowhage-induced itch. Single nerve fiber recordings in awake human subjects (microneurography), initially developed by Hagbarth and Vallbo (22) and adapted by Torebjörk and Hallin for C-fiber recordings (23), eventually demonstrated that a subgroup of mechanically insensitive (“silent”) C-nociceptors were particularly responsive to histamine and thus most likely convey histamine-mediated itch (24), whereas mechanically sensitive (“polymodal”) C-nociceptors were reliably activated by cowhage spicules (25) and thus, apparently mediate this form of histamine-independent itch (26). Notably, in addition to C-fibers also thinly myelinated A-delta nociceptors can be activated by cowhage spicules in monkeys (27). However, considering electrically induced itch (see Table 1) and the hitherto established profiles of transcutaneous high frequency (>50 Hz) stimulation with pulses of short duration (<2 ms), and bearing in mind the remarkably high electrical activation threshold of C-nociceptors compared to myelinated fibers (30), it remains open which primary sensory afferent nerve fibers explicitly had been activated in the investigations mentioned above (10–14, 17, 18). Both myelinated and unmyelinated fiber types are activated by high frequency electrical stimulation with rectangular pulses. The uncertainty of matching stimulus configuration to the activated nerve fiber class, however, is rather unsatisfying. This issue can be addressed by employing slowly depolarizing electrical stimuli of half-sine and sine wave shape delivered transcutaneously via small punctate electrodes that selectively activate C-fibers (33–36). Thereby, “polymodal” C-nociceptors are activated by a single 500 ms half-sine wave pulse responding with a current intensity dependent burst of action potential discharges (34). Notably, low-threshold unmyelinated tactile afferents also respond to that type of stimulus (34) but this fiber class is associated primarily with social touch rather than pain or itch processing (37, 38). In contrast, “silent” and “polymodal” C-nociceptors are activated by 4 Hz sine wave pulses (33, 36) of which “silent” nociceptors respond with one action potential per sinusoidal cycle compared to the discharge burst recorded from “polymodal” units (34, 36). Hence, the selection of a slowly depolarizing stimulation profile applied through pinpointed electrodes can be used to differentially activate nociceptor sub-types, which of course provoke in healthy human skin rather pain than itch but may change under pathologic conditions (see below).

**Table 1:** Parameters of electrical stimulation for experimental itch induction in humans.

**Wave form**	**Electrode configuration and dimension**	**Pulse duration**	**Stimulationfrequency**	**Stimulationintensity**	**Stimulation period**	**Optimal paramaters to elicit itch**	**Skin type and location**	**Fiber-class preferentially activated**	**Discharge pattern**	**References**
rectangular 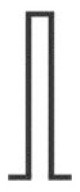	transdermal; 40G wire, 5 cm; 3,14 cm^2^ disc	n.d.	10 Hz	n.d.	several seconds	n.d.	healthy; dorsum hand and forearm	A-fibers	1 AP/pulse	(10)
	intra-cutaneous; pinpoint copper wire	5 ms	20 Hz	0.5–1.5 V	0.2 s	20 Hz; 5 ms	healthy	A- + C-fibers	1 AP/pulse	(11)
	transdermal; 5 cm^2^ gauze pad	10 ms	50 Hz	15–150 μA	>15 s	50–150 μA	healthy + AD non-lesional skin; volar wrist	A- + C-fibers	1 AP/pulse	(12)
	transdermal; 6 cm^2^ gauze pad	7 ms	10–40 Hz	supra-threshold	5 s	10 Hz; 7 ms	healthy	A- + C-fibers	1 AP/pulse	(13)
	transdermal; stainless steel wire 0.1 x 7 mm	0.08–8 ms	2–200 Hz	<0.12 mA	90 s	50 Hz; 2 ms; 0.05–0.1 mA	healthy + AD non-lesional skin; volar wrist	A- + C-fibers	1 AP/pulse	(14)
	transdermal; 3,14 cm^2^ disc	0.1 ms	50 Hz	1.6–6.4 mA	2 min	50 Hz; 0.1 ms; 6.4 mA	healthy; volar forearm	A- > C-fibers	1 AP/pulse	(17, 18)
half-sine 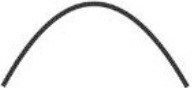	transdermal; pinpoint platinum wire 0.4 mm	500 ms	1 Hz	0.2–1 mA	500 ms	single pulse; 0.6–1 mA	AD lesional + non-lesional (volar forearm) skin	low-threshold and polymodal C-fibers	burst of APs/pulse	(28)
sine 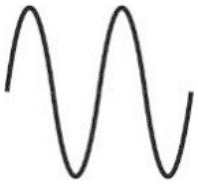	transdermal; 3,14 cm^2^ disc	200 ms and 0.0005 msper cycle	5–2000 Hz	2–140 μA	several seconds until itch threshold	5 Hz; 8–25 μA	healthy + AD/prurigo nodularis lesional + non-lesional	A- > C-fibers	1 AP/cycle	(29)
	transdermal; pinpoint platinum wire 0.4 mm	250 msper cycle	4 Hz	0.025–0.2 mA	1 min	4 Hz; 0.1–0.2 mA; continuous 30–60 s	AD lesional + non-lesional (volar forearm) skin	polymodal and silent C-nociceptors	1 AP/cycle	(28)

*Summary and references of the electrical stimulation protocols. Note that with a rectangular stimulation paradigm of 0.25 Hz and 0.5 ms pulse duration the transcutaneous electrical thresholds were >9 mA for C-nociceptor stimulation (electrode covering 30 mm^2^) (30). If higher current densities are provided at the stimulation sites by using pointed (1 mm diameter) or bipolar (2 mm length, 3 mm distance) pin electrodes (stimulation frequency 1–10 Hz) current intensities exceeding 5 mA are required for C-fiber activation (31, 32). Given the low intensities and high frequencies used in the protocols summarized, most likely myelinated A-fibers had been stimulated*.

*n.d. not determined*.

### Limitations

The stimulation paradigms of electrical rectangular pulses delivered to human skin (10–14, 17, 18) do not differentiate nerve fibers that contribute to histaminergic vs. non-histaminergic itch. This can be seen as a limitation of electrically evoked itch in experimental human studies when compared with chemical stimulation (e.g., histamine vs. cowhage spicules). The use of antihistamines during the electrical stimulation protocol might be suggested to overcome a possible lack of specificity and to rule out an unintentional activation of skin mast cells, but electrical activation of C-fibers should not require G protein-coupled receptors (e.g., histamine receptors). In contrast, itch caused by chemical stimulation involves signal transduction processes at the sensory endings and knowledge of these itch mediating pathways, indeed, may lead to the development of biologics [e.g., interleukin-receptor antibodies (39, 40)] and eventually successful anti-pruritic therapies. However, the number of chemical substances causing an itch indicate a plethora of key mediators and pathways being involved [see (41) for review]. Direct depolarization of primary afferent axons by electrical stimulation and subsequent induction of APs would skip these transduction processes, thereby bypassing any (perhaps disease-induced) changes in cutaneous pruriceptive nerve endings but of course also losing the opportunity to investigate them further. On the other hand, bypassing these potential changes at terminal nerve fiber endings through direct electrical axonal induction of APs might enable the unconfounded investigation of spinal itch circuits that might have been sensitized e.g., under pathologic conditions. Furthermore, the advantage of a precise timing of electrically evoked “itch onset” and “itch offset” can be used in combination with CNS imaging techniques (e.g., fMRI) providing a promising tool to amend human itch research.

### Electrically Evoked Itch in Atopic Dermatitis (AD) Patients

Based on the hypothesis that chronic inflammation might differentially modify neuronal excitability of skin afferents in pruritic skin, it is intriguing to investigate electrically induced itch in patients suffering from chronic itch. Until recently, only few studies explored this issue in atopic dermatitis (AD) patients (12, 14, 29). Edwards and colleagues demonstrated a faster response time of itch to different intensity levels of electrical stimulation. This was interpreted as a reduction of itch thresholds in AD, but unfortunately the authors did not record a dose-response for itch magnitude (12). The study by Ikoma and colleagues compared both histamine and electrically induced itch between healthy controls and AD patients (14). Read out parameters were electrically evoked itch, pain, tapping sensations, skin erythema and secondary areas of alloknesis, hyperknesis or punctuate hyperalgesia, but none of these measures were significantly different between healthy control subjects and AD patients (14). An explanation for these rather disappointing results might be that the peripheral primary afferent nerve fiber classes contributing to chronic itch in AD were not sufficiently activated by the electrical stimulation. Additionally, differences of sensation between healthy control subjects and patients might have been missed since AD patients were not stimulated in their itchy and/or eczematous skin. In another study, Pereira and colleagues stimulated peripheral nerve fibers of patients with chronic pruritus (AD and prurigo nodularis) by 5 Hz and 2 kHz transcutaneous electrical stimuli generated by a Neurometer® and delivered via a pair of gold electrodes (each 1 cm in diameter) attached the volar forearm skin (29). The Neurometer® is a device that produces a pure sine wave of up to 10 mA (42). It is commonly used to assess peripheral nerve fiber function and sensory symptoms in polyneuropathy patients (43). In six of the 78 investigated patients a sensation of itch was recorded upon 5 Hz stimulation and in two of 73 patients during 2 kHz stimuli (29). The relative small number of patients reporting electrically evoked itch might be due to the electrode configuration (diameter 1 cm) used for transcutaneous stimulation. High current densities facilitate the excitation of unmyelinated pruriceptors and therefore small pinpointed electrodes would be recommended for their recruitment (31). Also, the authors assessed the sensation of the patients at current perception thresholds, which may have hampered itch induction when compared with supra-threshold electrical stimulation.

Recent investigations demonstrated the differential activation of C-nociceptor subclasses by transcutaneous administration of a single electrical 1 Hz half-sine wave pulse (500 ms duration, current <1 mA) as well as a series of 4 Hz sine wave pulses (<0.4 mA), delivered through pinpointed electrodes (34, 36). Human psychophysics, skin erythema and sweat response measurements as well as compound action potential recordings *in vitro*, single nerve fiber discharge patterns monitored *in vivo* from pig saphenous nerve, and microneurography in humans thereby provided evidence that half-sine wave pulses activate primarily polymodal C-nociceptors. In particular, the C-fibers respond with a burst of APs in which the number of APs and their discharge frequency varies intensity-dependently to the single half-sine wave stimulus (34). In contrast, delivering 4 Hz sine wave pulses activates both, polymodal and “silent” C-nociceptors (36). Thereby, particularly “silent” C-fibers respond with a single AP per sine wave cycle revealing an activation pattern that is characterized by synchronized discharge at 4 Hz (34).

If slowly depolarizing sinusoidal stimuli of 4 Hz are applied continuously for a longer period (1 min), healthy human subjects report a gradual decline of the perceived burning pain sensation indicating a profound C-nociceptor accommodation to the electrical pulses. In contrast, neuropathic pain patients reported—particularly at their painful skin sites—an increased nociception without adaptation upon (supra-threshold) ongoing sine wave stimulation (36).

In a recent study, we used this electrical stimulation protocol of differential (“polymodal” and “silent”) C-nociceptor activation to investigate electrically evoked sensations in AD patients (28). Half-sine wave and sine wave stimuli were delivered to eczematous skin areas that had been particularly itchy before the investigation, and the corresponding sensation was compared to the electrically induced responses obtained from the patients' non-affected (if possible site matched) skin sites. Single half-sine wave pulses (500 ms, 0.2–1 mA) induced itch in about 30% of the patients. Delivering sinusoidal pulses for a duration of 2.5 s (4 Hz, 0.025–0.4 mA) caused itch in only three of 25 patients, all others mentioned discomfort or pain upon stimulation. Importantly, when the sine wave pulses were delivered continuously for 1 min (4 Hz, max. 0.2 mA) to eczematous skin sites, the number of patients reporting itch increased progressively the longer the stimulation lasted, resulting in about 50% of AD patients perceiving an itch at 1 min (all of them also perceived half-sine wave itch) (28). When stimulating non-affected skin sites only three patients reported itch. These results indicate that activation of both, “polymodal” and “silent” nociceptors, can evoke pruritus in AD and the duration of stimulation might be essential to induce it. Notably, an inter-individual variability in the sensation of itch upon electrical stimulation has to be considered as pruritus could be evoked in only a part of the AD patients. Possible reasons for variable responses might be individual differences in skin pathophysiology [for instance hypo- vs. hyper-innervation (44, 45)] or psychological stress status (46), which both might be a target for future experimental studies.

### Neuronal Pathways Mediating Itch

Currently, three models explain in general, why we feel an itch [reviewed in (47–49)]. In the mouse, dedicated pruriceptive sensory neurons exist and their activity is sufficient to produce pruritus (50, 51) along a so-called “labeled-line,” leading to the *specificity theory of itch*. This specificity theory was based on the use of itch-specific neuropeptides by pruriceptive sensory neurons or the involvement of spinal itch-specific transmission pathways (52, 53). In humans, sensory neurons that constitute a labeled-line for itch have not yet been described, as neurons activated by pruritogens also respond to algogenic stimuli. However, it was postulated that pruritogens induce lower AP frequencies or different AP patterns as compared to algogenic stimuli in these neurons, leading to the *intensity/pattern theory of itch* (54). Finally, focal application of pruciceptive or algogenic stimuli can lead to itch (55), potentially because the CNS interprets a heightened spatial contrast of peripheral input as itch, leading to the *spatial contrast theory of itch* (56, 57).

Independent of the exact coding mechanism of pruritus, it is a striking observation that AD patients often feel itch during stimulation of their eczematous skin upon a stimulus that is normally perceived as painful (28, 58). One likely mechanism of such a shift from pain to itch can be a changed central processing of the pruriceptive and/or nociceptive input in AD. Altered central processing on spinal and supra-spinal levels could facilitate transmission of pruriceptive information in ascending sensory circuits. A switch from an intense nociceptive stimulus (e.g., the injection of protons) in healthy human skin to an itch in AD patients has already suggested such a central sensitization process (58). In addition, a reduced descending inhibition of itch may be suggested in chronic pruritus, given that the effect of conditioned pain modulation was decreased (59) and transcutaneous electrical nerve stimulation (TENS, 100 Hz up to 26 mA) did not reduce acute itch sensation in AD (60). Intriguingly, cutaneous field stimulation applied to itchy skin areas of AD patients via 16 needle electrodes fixed at 2 cm intervals on a 4 × 4 cm flexible rubber plate by delivering 1 ms rectangular pulses at 4 Hz and up to 0.8 mA per electrode for 25 min (61) initially enhanced the intensity of itch in AD but significantly reduced it by about 25% after cessation for 1–5 h post-treatment (60). Changes in spinal circuits that determine the link between peripheral sensory input and the output of the different classes of spinal projection neurons ascending into the brain could also explain the switch from pain to itch observed in AD patients when delivering sine wave pulses to the eczema sites for longer duration. However, slowly depolarizing electrical stimuli activate specific peripheral afferent nerve fiber classes and can evoke itch only inside the eczematous skin in some (but not all) AD patients. This indicates that the stimulation of “polymodal” and “silent” nerve fibers—alone or in combination—contributes to a peripheral mechanism for the induction of itch that takes place in AD patients in addition to potential central mechanisms.

### Parameters Influencing Electrical Nerve Fiber Activation in the Skin

Excitability of sensory nerves to transdermal electrical stimulation depends—in principle—on three major cellular characteristics of a given nerve fiber: (a) the exact geometry of the nerve ending in the skin; (b) the membrane characteristics determining the extent of local depolarization upon electrical stimulation; (c) the encoding of the depolarization into discharges of single APs or bursts. All of these characteristics may be modified particularly by local inflammatory processes in eczematous skin and therefore could contribute to the observed itch upon normally painful electrical stimulation (28).

- In various skin diseases, including AD, epidermal innervation patterns change. However, the direction of this change is under debate, potentially due to different quantification methods of epidermal nerve fiber density. Initially, hyper-innervation of eczematous skin was proposed as a structural rearrangement causing chronic pruritus in AD (45, 62, 63). However, more recently, investigations in bigger patient cohorts as well as the use of various microscopic methods that allow imaging of large dermal volumes point to an epidermal hypo-innervation of eczematous AD skin (59, 64, 65). In line with this finding, we did not find decreased sensory thresholds to sine wave stimulation between eczematous/itchy and un-affected skin of AD patients as well as between AD and control subjects (28), as would be predicted from hyper-innervated skin. However, due to the lack of established markers for human pruriceptive nerve fibers—the mentioned studies used the pan-fiber marker protein gene product 9.5 (PGP9.5)—the significance of such epidermal innervation changes for electrical induction of pruritus in AD patients remains unclear and needs further investigation. Epidermal thickening, especially prevalent in eczematous AD skin, adds an additional layer of complexity as it might increase the distance between the most superficial nerve fibers and the transdermal stimulation electrodes as well as the length and axonal branching pattern of the nerve terminals, both of which might influence their excitability (65, 66).
- Most ion channels with established roles in the transduction of natural nociceptive stimuli, including the polymodal transient receptor potential (TRP) superfamily members TRP vanilloid 1 (TRPV1) and ankyrin 1 (TRPA1), are only weakly voltage dependent, limiting their contribution to electrically induced depolarization (67, 68). However, TRPV1 has been found to interact with voltage sensitive potassium channels (69) and might therefore also modulate neuronal excitability. Moreover, the voltage dependence of TRP channels is highly plastic in disease, thereby contributing to inflammatory pain states (70, 71). Interestingly, TRPV1- and TRPA1-expression was elevated in eczematous AD skin, an effect attributable to an increased expression per cell, as the number of TRPV1/TRPA1-immunopositive nerve fibers was unchanged (72). Thus, under inflamed conditions, in addition to a potential sensitization of their voltage dependence, the overexpression of TRPA1 and TRPV1 might increase the depolarizing effects of sinusoidal stimulation and thereby facilitate neuronal discharge. A potentially increased recruitment of TRPV1/TRPA1^+^ fibers upon sinusoidal stimulation in AD and the accompanied perception of itch would be in line with a previous observation, in which the administration of protons (known to activate TRPV1) evoked itch in lesional and healthy appearing skin of AD patients, but burning pain in control subjects (58).
- Receptor potentials are encoded into trains of APs at the so-called spike initiation zone. The position of this zone can be dynamically moved closer to the receptive endings under inflammatory conditions, thereby facilitating encoding of the receptor potential in APs as shown in corneal nociceptors in mouse (73). Such modulation of axonal excitability might also occur in inflamed human skin but has not yet been studied. With the advent of next-generation sequencing techniques, expression changes of ion channels in eczematous skin of AD patients have been investigated, using a dermal punch biopsy as input material (72, 74, 75). This bulk analysis, though powerful, also has some caveats. For instance, differences in the cellular constituents of the biopsy involuntarily lead to differences in gene expression. In line with this notion, genes selectively expressed by invading leukocytes show high overexpression in eczematous skin (72, 74, 75). Expression changes in nerve fibers are particularly hard to detect in punch biopsies, as they make up only a minute amount of the tissue's total RNA, are not specifically targetable by current single cell transcriptomic approaches (76) and only refer to axonally transported RNA. These problems also preclude to link global transcriptomic changes to specific nerve fiber classes, which is particularly warranted, given that sinusoidal transcutaneous electrical stimuli preferentially activate C-fibers (35, 36). However, despite these technical difficulties, one study found the voltage-gated sodium channels Nav1.3, Nav1.7, and Nav1.9 to be overexpressed in eczematous skin of AD patients and, importantly, this overexpression correlated with pruritus severity (72). Assuming that neurons were the only cell type expressing voltage-gated sodium channels in the skin, the authors concluded that their overexpression in the eczematous lesions might indicate a sensitized state, which could potentially explain increased responsiveness to slowly depolarizing sine wave stimulation. Particularly Nav1.7 might be a target of axonal hyper-excitability, as this channel can amplify slow depolarizations (77) by producing so called ramp currents that are based on the channels slow closed-state inactivation kinetics. As the time course of depolarization during the 4 Hz sinusoidal stimulation fits to such ramp currents, Nav1.7 might facilitate the electrically induced activation of axons (78, 79) and possibly contributes to the itch in AD patients.

In addition to voltage-gated sodium channels, potassium channels are major determinants of nociceptive discharge patterns. Indeed, Esaki and colleagues found a member of the Kv1 family of voltage-gated potassium channels (Kv1.3, encoded by KCNA3) to be upregulated in eczematous skin of AD patients (74). Specifically, this upregulation was only detectable in the dermis (72, 74). As Kv1 channels are involved in limiting the maximal AP frequency in nociceptors (80), it is possible that the observed upregulation of Kv1.3 limits electrically induced nociceptive input to the spinal cord that would have inhibited the spinal transmission of pruriceptive information to the brain under normal conditions (81).

### Temporal Electrical Stimulation Patterns and Their Role in Itch Induction in AD Patients

The duration of electrically evoked ongoing primary afferent nerve fiber stimulation seems to play a pivotal role for itch induction in AD patients. Our recent studies demonstrated that eliciting pain or itch in patients does not only depend on the stimulation of the specific nerve fiber classes, but is also dependent on the actual duration of the stimulation (28, 36). In healthy human subjects, sinusoidal electrical stimulation evokes pain that adapts substantially upon ongoing electrical stimulation with 4 Hz sine waves (36). By contrast, the same stimulation protocol induced increasing pain in chronic pain patients, particularly at neuropathic painful skin sites (36). Similarly, we observed progressively increasing pruritus in AD patients when stimulating with ongoing sinusoidal stimulation, indicating that in both groups of patients the nerve fiber classes activated by the electrical stimulation appear resistant to adaptation. This kind of activity-dependent change of pruriceptor or C-nociceptor excitability upon ongoing stimulation is important to differentiate from acute activation thresholds of these nerve fibers to a single stimulus, in particular when trying to link it to a potential mechanism contributing to chronic itch (or chronic pain) that is based on spontaneous discharge of C-fibers lasting for prolonged periods. However, prolonged neuronal input has also implications for the spinal itch processing as sustained peripheral neuronal input may be required to facilitate spinal itch transmission. Such spinal circuit changes involve, for instance, the activation of gastrin-releasing peptide (GRP) receptor neurons (82). Zeilhofer and colleagues showed that the release of GRP from spinal interneurons is a prerequisite for the transmission of pruriceptive information to higher itch centers in the brain, which, importantly, requires ongoing peripheral input to induce several periods of burst-like activity in spinal GRP^+^ neurons (82). A sufficient interaction of GRP^+^ and GRP-sensing neurons, possibly triggered during our ongoing sinusoidal stimulation of primary afferent neurons, may have initiated itch in AD patients. On the other hand, our electrically induced neuronal input could also inhibit spinal itch processing via GABAergic (83) or glycinergic (84) signaling. Thus, long-lasting slowly depolarizing electrical stimulation protocols that cause itch in a sub-group of AD patients probably indicate facilitated spinal processing and/or weaker inhibition of itch and might therefore help to identify those patients that benefit from spinally acting antipruritic therapy.

### Perspectives

Slowly depolarizing transcutaneous electrical stimulation provides functional assessment of both, pruriceptors (e.g., in AD patients) and nociceptors (e.g., in neuropathic pain patients). The precise electrical protocols, including the temporal profile of stimulation, are of particular importance to generate peripheral input from different classes of C-nociceptors and pruriceptors. Furthermore, the advantage of a controlled pruriceptive/nociceptive stimulus onset and offset can be exploited in various explorative directions, for instance when combining the stimulation profile with other techniques (like EEG or fMRI) to investigate the human brain. Future research may help to link specific functional attributes of electrically evoked responses and structural changes to the patient's symptoms. This would facilitate a better understanding of the peripheral and also central processing of pruriceptive and nociceptive inputs in general.

## Author Contributions

All authors contributed to the article and approved the submitted version.

## Conflict of Interest

The authors declare that the research was conducted in the absence of any commercial or financial relationships that could be construed as a potential conflict of interest.
